# Gastrointestinal Motility, Mucosal Mast Cell, and Intestinal Histology in Rats: Effect of Prednisone

**DOI:** 10.1155/2017/4637621

**Published:** 2017-09-19

**Authors:** Maysa Bruno de Lima, Loyane Almeida Gama, Andrieli Taise Hauschildt, Denize Jussara Rupolo Dall'Agnol, Luciana Aparecida Corá, Madileine Francely Americo

**Affiliations:** ^1^Federal University of Mato Grosso, UFMT, Barra do Garças, MT, Brazil; ^2^São Paulo State University, UNESP, Botucatu, SP, Brazil; ^3^Alagoas State University of Health Sciences, UNCISAL, Maceio, AL, Brazil

## Abstract

Our aim was to verify the effects of prednisone related to gastrointestinal motility, intestinal histology, and mucosal mast cells in rats. Two-month-old male Wistar rats were randomly assigned to control group (vehicle) animals receiving saline 0.9% (*n* = 7) or treated orally with 0.625 mg/kg/day of prednisone (*n* = 7) or 2.5 mg/kg/day of prednisone (*n* = 7) during 15 days. Mast cells and other histologic analyses were performed in order to correlate to gastric emptying, cecum arrival, and small intestine transit evaluated by Alternating Current Biosusceptometry. Results showed that prednisone in adult rats increased the frequency of gastric contractions, hastened gastric emptying, slowed small intestinal transit, and reduced mucosal mast cells. Histologically, the treatment with both doses of prednisone decreased villus height, whereas longitudinal and circular muscles and crypt depth were not affected. These findings indicate an impairment of intestinal absorption which may be linked to several GI dysfunctions and symptoms. The relationship between gastrointestinal motor disorders and cellular immunity needs to be clarified in experimental studies since prednisone is one of the most prescribed glucocorticoids worldwide.

## 1. Introduction

Glucocorticoids and their derivatives are the most prescribed synthetic drugs in clinical practice due to their large immunomodulatory activity [1]. Over the last 20 years, more than 30% of the general population in the US and in the UK received systemic glucocorticoid therapy [2–4]. In this scenario, prednisone stands out in the treatment of numerous inflammatory and autoimmune diseases, and as a part of immunosuppressive regimens after transplantation [3, 5]. Even though the efficacy of glucocorticoids is indisputable, they are associated with several adverse effects linked to long term use and/or high dose administration [1, 4]. Recent studies indicate that, in contrast with long term use, complications regarding short term use are much less understood, and data is insufficient to attend clinical practice guidelines [6]. However, studies towards evaluating side effects of prednisone on the gastrointestinal (GI) tract are controversial, although absorption surface, cellular transport, motility, and pH may modify its pharmacokinetics [7].

For several gastrointestinal diseases, the role of the mucosal immunity is currently being explored [8]. Due to its anti-inflammatory and immunosuppressive activities, prednisone can induce suppression in the subpopulation of immune cells in the intestinal mucosa [9]. Intestinal mast cells (MC) have an important role in host defense against microbes, mucosal regulatory functions, epithelial cells secretions, and smooth muscle contraction and peristalsis [10]. Also, chemical mediators released by activated mast cells can interact with enteric neurons and trigger physiological changes in GI tract, contributing to visceral hypersensitivity and dysmotility [11].

Adverse effects of prednisone are well-documented for several systems and/or for specific diseases. In mice pulmonary tissue, treatment with prednisone was able to reduce the mucosal mast cell transendothelial migration [12]. However, the effects of prednisone on GI histophysiological parameters and motility had not yet been documented or even neglected. In this context, novel studies could offer additional insights into normal physiology and the alterations caused by short term use of prednisone.

Noninvasive techniques such as Alternating Current Biosusceptometry (ACB) are essential to evaluate GI motor functions, including gastric contractility [13], gastric emptying, and intestinal transit [14] in physiological conditions expressing more accurate results [14, 15]. New and harmless studies focusing on the relationships between the immune system, intestinal mucosa, and motility contribute towards increasing the knowledge to support the short term use of glucocorticoids for treatment of gastrointestinal diseases [1, 16]. Hence, the aim of this study was to investigate the effects of prednisone regarding gastrointestinal motility, intestinal histology, and mucosal mast cells in rats.

## 2. Materials and Methods

### 2.1. Animals and Experimental Groups

Male Wistar rats (250–300 g) were maintained in controlled conditions of temperature (22 ± 3°C), humidity (60 ± 5%), and 12-hour light/dark cycle with access to commercial chow (Purina®) and filtered water ad libitum. All experimental procedures were approved by the Ethics Committee on Animal Research from Federal University of Mato Grosso (protocol number 23108.049862/13-3) and followed the Guidelines for Ethical Conduct in the Care and Use of Experimental Animals.

Animals were randomly assigned to control group, in which animals received only vehicle (0.9% NaCl) (*n* = 7), and treated group, in which animals received 0.625 mg/kg/day of prednisone (*n* = 7) or 2.5 mg/kg/day of prednisone (*n* = 7). Vehicle or prednisone treatments were administered orally during 15 consecutive days, always at the same time period. All analysis were performed at the end of the administration of vehicle or prednisone.

### 2.2. Alternating Current Biosusceptometry (ACB)

Magnetic monitoring of GI transit and contractility was performed employing the ACB technique (Br4-Science®, Brazil) [13, 14]. ACB sensor measures the magnetic flux variation between excitation and detection coils through lock-in amplifiers. Signals generated by magnetic materials in response to an applied magnetic field are detected and the signal intensity depends on the amount of magnetic material and the distance between the sensor and sample. In this study, ferrite powder (MgZnFe_2_O_3_, Imag, Brazil) was used as a nonabsorbable magnetic marker incorporated to laboratory chow. Detailed technical information has been reported earlier [13, 15].

### 2.3. Gastrointestinal Transit

After fasting overnight, animals were fed with the magnetically marked chow (1.6 g laboratory chow blended with 0.4 g ferrite powder). ACB sensor was placed on the abdominal surface and the maximum magnetic signal intensity value for both stomach and cecum projection (based on anatomical references) was recorded. Subsequent measurements were performed in awake rats upon those same points at regular 15-min intervals for at least 5 h [14].

### 2.4. Gastric Contractility

For GI transit measurements, the animals were anesthetized with 75 mg/kg ketamine (Cetamin®, Syntec, Brazil) plus 2.5 mg/kg acepromazine (Acepran® Vetnil, Brazil), intraperitoneally. Animals were then laid in supine and the ACB sensor was placed on the stomach surface towards recording the magnetic signals continuously for 30 minutes at a sampling rate of 20 Hz, by using a multichannel recorder (MP100 System; BIOPAC, Santa Barbara, CA, USA) [13].

### 2.5. Sample Collection

After gastric contractility measurements, animals were killed by anesthetic overdose consisting of 240 mg/kg ketamine plus 45 mg/kg xylazine chlorhydrate solution (Xilazin®, Syntec, Brazil) administered intraperitoneally. Immediately after the anesthetic overdose, the animals were decapitated in order to collect blood and tissues for analysis.

### 2.6. Blood Cell Count

Blood cell count for both control and treated animals groups was carried out by two different methods. Firstly, to analyze the total number of leukocytes, blood samples collected in EDTA-coated tube were diluted 1 : 20 in Turk solution, counted using a Neubauer chamber, and the results were expressed in 10^3^/mm^3^. Secondly, to differential cell counts, a total of 100 cells were obtained using a blood smear, and the slides were stained with panoptic (Instan-prov, Neuprov®). Cell populations were differentially counted based on the morphological features and the results were presented in absolute values (10^3^/mm^3^) [17].

### 2.7. Histological Analysis

Tissue samples from duodenum were fixed in methacarn (60% methanol, 30% chloroform, and 10% glacial acetic acid), dehydrated in alcohol series, cleared in xylene, and embedded in paraffin. Semiserial 4 micrometers (*μ*m) sections (microtome HM-355S Automatic Microtomes Thermo Scientific) were stained with toluidine blue 0.5% and hematoxylin and eosin (HE). Toluidine blue 0.5% staining sections were used to identify mast cells (MC) in the intestinal mucosa, since MC granules display metachromatic staining after uptake of toluidine blue dye. For each rat, sections of duodenum were randomly selected. Twenty well-oriented villus-crypt units (VCU) were examined per animal and expressed as mucosal MC per VCU [18]. HE staining was used to morphometric measurement of villus height, crypt depth, and thickness of the circular and longitudinal muscle layers. Images were captured on an optical microscope (Zeiss, Germany) coupled to a high-resolution camera (AxioCam ERc5s, Zeiss, Germany) and analyzed using the ZEN Blue Software 2011 (Zeiss, Germany) [19]. Analyses concerning MC mucosal number and morphometric measurement were blinded to avoid bias.

### 2.8. Data Analysis

GI transit data was analyzed in Origin® and statistical moments were calculated in order to obtain the Mean Gastric Emptying Time (MGET), as the time *t* (min) in which the average amount of magnetic material has been emptied from the stomach, weighted by the area under the emptying curve; the Mean Cecum Arrival Time (MCAT) defined as the time *t* (min) in which there was an increase in the average amount of magnetic meal that reached the cecum, weighted by the area under the curve between the arrival at the cecum and the cumulative maximum value; and the Mean Small Intestine Transit Time (MSITT) which was determined by the difference between MCAT and MGET [15, 20].

Gastric contractility signals were analyzed in Matlab by visual inspection and subsequently, Fast Fourier Transform (FFT) was applied. The highest peak of frequency for each FFT was determined as the gastric dominant frequency and the lowest was the intrinsic noise of the signal. Frequencies were expressed in hertz (Hz) and then converted to cycles per minute (cpm). Amplitude of contraction was determined by the ratio between the intensity of gastric peak (*P*) and noise peak intensity (*P*′) and expressed in decibels (dB) as follows:* A* = 10 log 10 (*P*/*P*′) [21].

### 2.9. Statistical Analysis

All results were expressed as mean ± standard deviation (SD) and analyzed by ANOVA, followed by Tukey. Pearson correlation coefficient (*R*) was applied to evaluate the relationship between intestinal variables. Only coefficients above 0.80 were considered significant. Differences were considered significant at a *p* value < 0.05.

## 3. Results

The treatment with prednisone reduced the total leukocytes number and differential count compared with control (vehicle) group, confirming the immunosuppression in both doses, as expected (Table 1).

**Figure 1:**
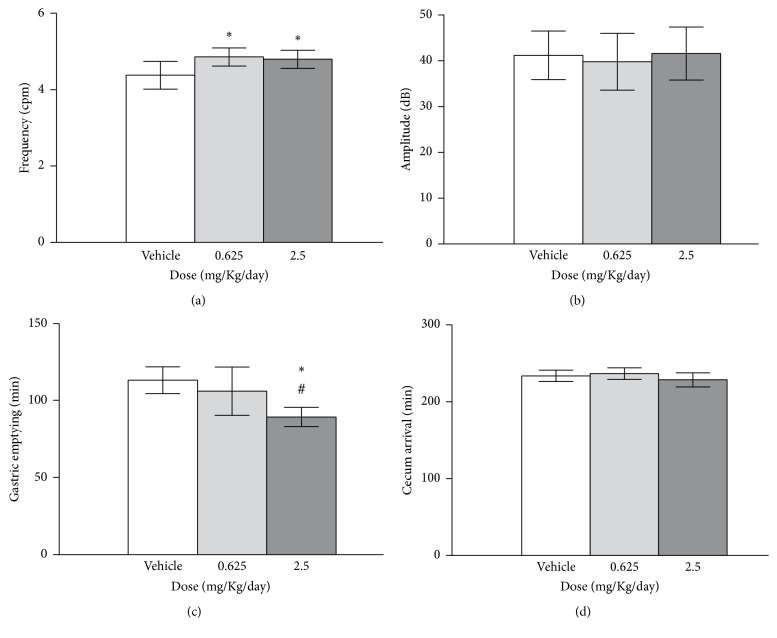
Frequency of gastric contractions (a), amplitude of contractions (b), gastric emptying (c), and cecum arrival (d) were determined for all groups. For comparison between groups: ^*∗*^*p* < 0.05 versus vehicle; ^#^*p* < 0.05 versus 0.625 mg.

Treatment with prednisone, in both doses, has increased (~4.8 cpm) the frequency of gastric contractions compared with the vehicle (4.4 ± 0.4) (Figure 1(a)) whereas the amplitude of contraction showed nonsignificant changes between groups (Figure 1(b)). Animals treated with 2.5 mg/Kg of prednisone had accelerated gastric emptying compared with vehicle (Figure 1(c)). Cecum arrival time (Figure 1(d)) was not different among groups.

**Figure 2:**
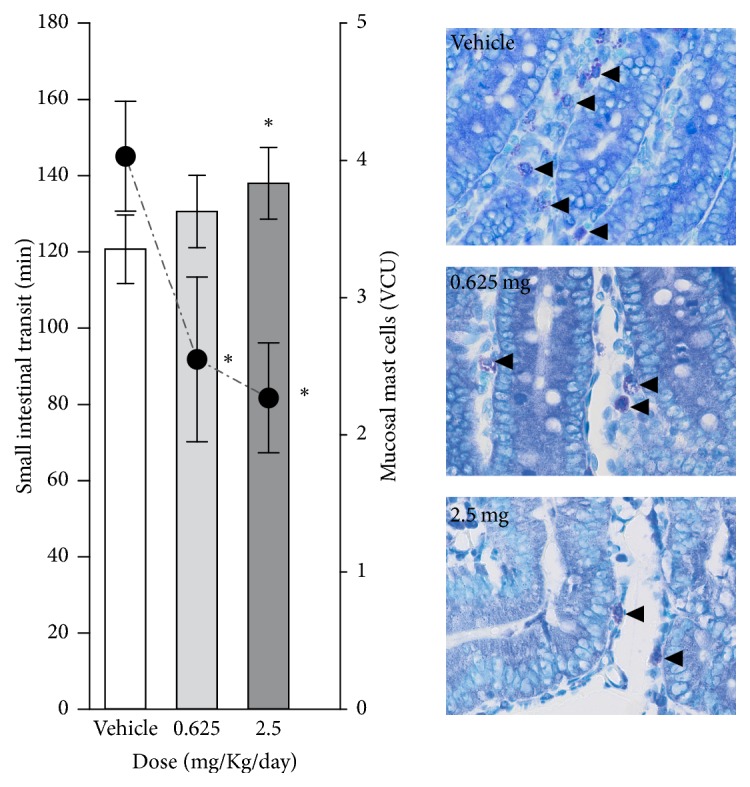
Small intestinal transit, mucosal mast cell, and correlation between them were determined for all groups. Arrows indicate mucosal mast cell in the histological examples. For comparison between groups: ^*∗*^*p* < 0.05 versus vehicle.

As shown in Figure 2, the treatment with 2.5 mg/Kg of prednisone slowed intestinal transit compared with vehicle. Regarding the mast cell analysis, the prednisone in both doses reduced the mucosal MC number. Also, there was a significant positive correlation between small intestinal transit time and MC from mucosa (above* R* = 0.8) after prednisone treatment (Figure 2). In control conditions, there was no correlation between these parameters.

**Figure 3:**
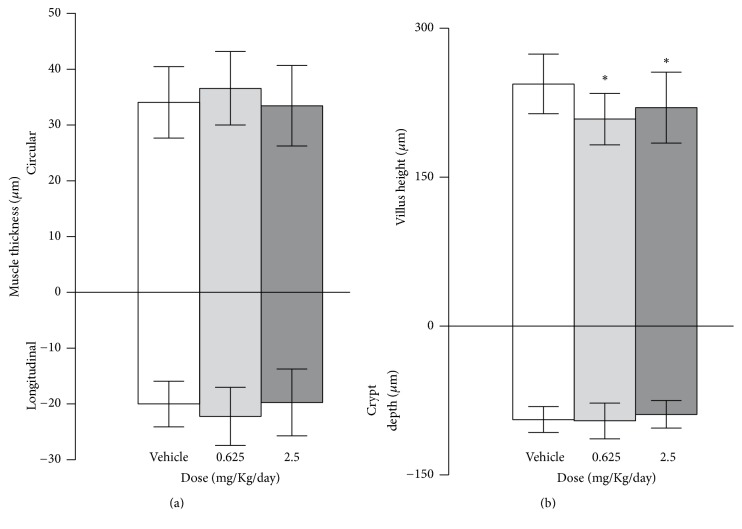
Muscle thickness (longitudinal and circular) (a) and villus height and crypt depth (b) were calculated for all groups. For comparison between groups: ^*∗*^*p* < 0.05 versus vehicle.

Muscular layers and crypt depth had no changes after prednisone treatment, while villus height decreases for both treated groups compared with the vehicle (Figure 3).

## 4. Discussion

Our results indicate that the short term use of prednisone modified the gastrointestinal function and the morphological structure. Increased frequency of gastric contraction, accelerated gastric emptying, slowed intestinal transit, and decreased number of mucosal mast cells and villus height were observed.

Gastric emptying is a limiting step in the absorption of orally administered drugs and nutrients [22]. In previous studies, we already showed a hastened gastric emptying in male rats treated with prednisone after intermediate dose [23]. As prednisone was able to decrease the villus height in duodenum, the association of both data suggests a change in gastrointestinal absorption which may compromise the uptake of essential nutrients [24] and also the bioavailability of drugs since intraluminal environment has a great influence on the performance of the dosage forms administered orally [25, 26].

As expected, there was a decrease in leukocytes and mucosal mast cell after prednisone treatments [27]. Studies have shown the glucocorticoids cause apoptosis of lymphocytes in rodents [28] and also reduced mast cells in lungs [29, 30]. Prednisone-treated mice had reduction of the inflammatory response in allergic pulmonary inflammation followed by the decrease of mast cell influx to the mucosa [12]. However, the effects of prednisone on intestinal mast cells had not yet been documented.

Oral prednisone therapy results in relevant time- and dose-dependent toxicity in diverse systemic diseases [31] and it is related to several side effects. In healthy condition, these alterations may have no clinical relevance; but it can be relevant in sick, geriatric, or debilitated patients [32]. Under specific conditions, for example, in lupus, prednisone has been consistently associated with increased irreversible damage and increasing rate of morbidity and mortality [31, 33]. Several studies have demonstrated adverse gastrointestinal effects after short term administration of high doses of methylprednisolone and dexamethasone [32, 34–36]. Nevertheless, clinical trials comparing high (>30 mg and up to 100 mg per day) versus low (≤7.5 mg/day) or equivalent doses of prednisone are scant [31].

Quantification of adverse effects related to doses of glucocorticoids is still challenging, especially considering patients rarely receiving glucocorticoids as monotherapy [33]. The use of prednisone associated with other drugs complicates the assessment of their individual effects, since synergistic and/or antagonistic drug interactions can occur allowing prednisone to be considered harmless [37]. Doses < 7.5 mg of prednisone daily seem to minimize adverse effects, but the dose below which treatment can be considered safe has not been defined [31]. However, even low doses of glucocorticoids (e.g., as high as 6 mg/day) can be associated with organ damage [33, 38].

Studies focusing on the effects of prednisone on gastrointestinal motility and the relationship to the local immunity are limited [39, 40]. Our study showed a strong positive correlation between intestinal transit time and the number of mucosal mast cells after prednisone treatment at both doses (Figure 2). Positive correlation between the number of mucosal mast cells and intestinal permeability in patients with diarrhea-predominant irritable bowel syndrome was also observed by Lee and collaborators [41]. Mast cell activation in intestinal mucosa releases mediators such as histamine, chymase, and prostaglandin which regulate the permeability [42, 43] and the protection maintained by the integrity of luminal epithelial barrier [44].

Besides regulation of the permeability, the enteric nervous system and cytokines such as IL-4 and IL-13 appear to be involved in the relationship between the number of mast cells and motor activity [10, 45]. Mast cells can be found in close spatial contact with interstitial cells of Cajal (ICC); however, studies focused on their distribution in rodent intestinal muscularis externa and association with motor function are lacking [46]. Propulsion of intestinal contents needs to be at an optimum rate to prevent complications arising from stasis, such as intestinal bacterial overgrowth [47]. Intestinal dysmotility can be associated with nausea and vomiting, bloating, or even visible distension [48, 49].

The evaluation of gastrointestinal transit time, gastric emptying, and contractility is fundamental to understand the effect of drugs and the interaction with the gastrointestinal tract. Strain-gauge (SG) transducers implanted in laboratory animals and manometric tubes employed in several species, including humans, are often used. Nevertheless, both are invasive approaches, requiring surgery and uncomfortable catheter insertion, respectively [50]. Manometry records only pressure waves that occlude the lumen and, unlike SG, is unable to detect all contractions [13]. On the other hand, ACB allows evaluating GI transit* in vivo* embracing all the interference of gut hormone levels and, most importantly, on an intact enteric nervous system and gastrointestinal mucosal immunity.

## 5. Conclusion

Prednisone administered to rats increased the frequency of gastric contractions, hastened gastric emptying, slowed small intestinal transit, reduced mucosal mast cells, and decreased villus height. These findings indicate an impairment of intestinal absorption which may be linked to several GI dysfunctions and, thereafter, symptoms, which need to be clarified since prednisone is one of the most prescribed glucocorticoids worldwide. Noninvasive techniques such as ACB are promising tools towards evaluating the side effects of time course treatments on GI motility.

## Figures and Tables

**Table 1:** Total leukocytes and differential cell counts after vehicle or prednisone treated rats with 0.625 mg/Kg/day and 2.5 mg/Kg/day, respectively.

	Vehicle	0.625 mg/Kg	2.5 mg/Kg
Total leukocytes	4.41 ± 0.26	2.43 ± 0.32^*∗*^	2.49 ± 0.06^*∗*^
*Lymphocytes*	2.83 ± 0.11	1.37 ± 0.36^*∗*^	1.37 ± 0.27^*∗*^
*Neutrophils*	1.40 ± 0.11	0.94 ± 0.18^*∗*^	1.01 ± 0.27^*∗*^
*Monocytes*	0.13 ± 0.07	0.09 ± 0.04	0.09 ± 0.03
*Eosinophils*	0.04 ± 0.0	0.02 ± 0.01^*∗*^	0.01 ± 0.01^*∗*^

Cell counts expressed in 10^3^/mm^3^; ^*∗*^*p* < 0.05 versus vehicle.
